# Pneumococcal meningitis in a patient with severe COVID-19 on dexamethasone and tocilizumab: A case report

**DOI:** 10.1016/j.idcr.2023.e01727

**Published:** 2023-03-03

**Authors:** Yoshiaki Murayama, Tomohiko Ishimine, Mikio Sasano, Takafumi Todaka, Takashi Matsumoto, Taiga Shimabukuro, Risa Yonaha

**Affiliations:** a Department of Respiratory Medicine, Nakagami Hospital, 610 Noborikawa, Okinawa city, Okinawa 904-2142, Japan; b Department of Critical Care Medicine, Nakagami Hospital, 610 Noborikawa, Okinawa city, Okinawa 904-2142, Japan

**Keywords:** COVID-19, SARS-CoV-2, Tocilizumab, Streptococcus pneumoniae, Meningitis, Secondary-infection

## Abstract

Although various therapeutic agents have been tried for coronavirus disease-2019 (COVID-19) and evidence has accumulated, the risk of secondary infection is increased by underlying disease and immunosuppressive drugs. We report a case of pneumococcal meningitis in a patient with severe COVID-19 who was receiving dexamethasone and tocilizumab. The patient's symptoms improved with appropriate diagnosis and antimicrobial therapy, and she fortunately returned to society without any neurological sequelae of meningitis.

## Introduction

Various therapeutic agents have been tested against COVID-19, evidence has accumulated, and guidelines [1] are being developed. However, immunosuppression caused by therapeutic agents and underlying diseases has been observed in some cases, resulting in secondary infections [2], [3]. Although antimicrobial therapy is not required at admission in many cases, secondary infection becomes a problem when patients are immunosuppressed due to therapeutic agents for COVID-19 or underlying disease.

In this report, we describe a case of pneumococcal meningitis in a patient with severe COVID-19 who was receiving dexamethasone and tocilizumab. The patient's symptoms improved with appropriate diagnosis and treatment with antimicrobial agents, and fortunately, she was able to return to the community without any neurological sequelae of meningitis.

## Case presentation

**Fig. 1:**
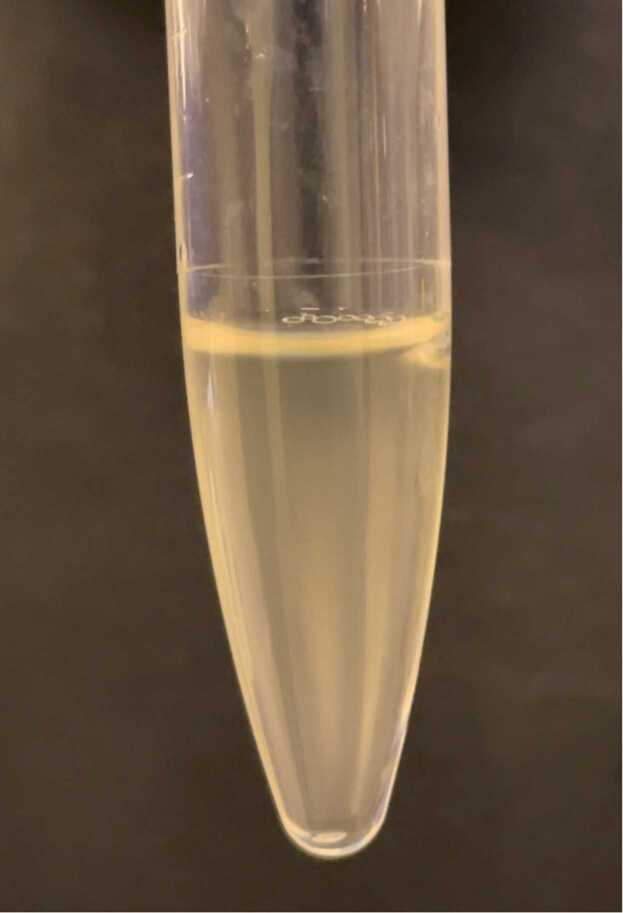
Cerebrospinal fluid (CSF) The CSF was grossly thick.

The patient is a 72-year-old woman. She presented to the outpatient department of internal medicine with a chief complaint of fever and dyspnea that persisted for 3 days. Her daughter and husband had been diagnosed with COVID-19. She had bronchial asthma, hypertension, and type 2 diabetes mellitus (HbA1c 6.6%). She had no smoking history. The SARS-Cov-2 antigen test and SARS-Cov-2 PCR test were positive, and a diagnosis of COVID-19 was made. On admission, the patient was conscious and clear (Glasgow Coma Scale: E4V5M6). She had no headache on admission or during the clinical course. She had a temperature of 38.0℃38.0 ℃ (axillary), respiratory rate of 24 breaths/min, and hypoxemia with SpO2 of 88% (in room air). Respiratory investigation revealed diffuse bilateral coarse crackles, but no wheeze. Physical examination was otherwise normal. Laboratory tests showed a white blood cell count of 21,240/µL (74% neutrophils, 10.9% lymphocytes) (reference range (RR), 1000–4800/µL). Additionally, serology showed revealed elevated lactate dehydrogenase level of 292 IU/L (RR 124–222 IU/L), C-reactive protein levels of 7.61 mg/dL (RR < 0.3 mg/dL), and elevated creatinine level of 1.61 mg/mL (RR < 1.0 mg/dL). Urinalysis was normal. Chest CT showed bilateral pneumonia. Dexamethasone 6 mg/day and Remdesivir 100 mg/day (initially 200 mg/day) were started on the first day of treatment for COVID-19 moderate disease with oxygen demand. Oxygen demand was initially 4 L/min on the oximizer, but increased during the night on the first day, and oxygen administration by High Flow Nasal Cannula (FiO2 0.65, Flow 50 L/min) was started. On the fourth day of the illness, oxygen demand increased, requiring FiO2 of 0.8, and the patient was admitted to the ICU and endotracheal intubation was performed. On the same day, a single dose of Tocilizumab 8 mg/kg/day was administered, and Remdesivir was discontinued. The patient remained in a lull state until the ninth day of treatment when fever developed. The patient's level of consciousness was difficult to assess because she was under sedation. Physical examination and abdominal ultrasonography revealed no heat source. Microscopic pyuria was observed, but since there were no change of other vital sign, blood and urine cultures were obtained and the patient was monitored for a febrile form. On the 10th day, blood culture was positive for 6/6 Gram-positive cocci (streptococci). Considering the possibility of catheter infection, the catheters (central venous catheter, arterial line, and venous line) were replaced and Vancomycin 1.5 g/day was started. On the 11th day, the Gram-positive cocci was identified as streptococcus pneumoniae. Since streptococcus pneumoniae was the causative organism of bacteremia, catheter infection was unlikely, and we considered the possibility of meningitis. Although the patient did not have rigidity of the neck, a cerebrospinal fluid (CSF) puncture was performed, and the CSF was grossly thick (Fig. 1). The CSF examination showed an increased cell count (Number of cells 2998/µL, (Mono 6.8%, Poly 93.,2%), Protein 468.5 mg/dL, Glucose 24 mg/dL.), and a diagnosis of bacterial meningitis was made. Gram staining of CSF and CSF culture were negative, possibly due to post-antimicrobial administration. The patient was started on a meningitis dose of ceftriaxone 4 g/day and vancomycin 2 g/day. An additional blood culture taken on the 12th day was positive for Gram-negative rods in 2/2 sets and considering the possibility of Extended Spectrum beta Lactamase (ESBL-s)-producing organisms, antimicrobial escalation from Ceftriaxone to Meropenem 6 g/day was performed. The Gram-negative rods were Klebsiella spp. The Gram-negative rod was found to be Klebsiella pneumoniae, which was de-escalated to 3rd cephem after confirming susceptibility. The main pathogenesis was meningitis caused by Streptococcus pneumoniae, but the possibility that Klebsiella pneumoniae was the pathogen of meningitis could not be ruled out, and antimicrobial agents were administered for a total of 21 days. On the 13th day, we confirmed that the patient's level of consciousness had improved, and we performed a spontaneous awakening trial and a spontaneous breathing trial before performing extubation. After extubation, the patient's respiratory status and level of consciousness stabilized, and fortunately, no sequelae of meningitis were observed. On the 34th day, the patient was transferred to a rehabilitation hospital. After leaving the rehabilitation hospital, she returned to society.

## Discussion

We report a case of pneumococcal meningitis in a patient with severe COVID-19 on dexamethasone and tocilizumab. Dexamethasone has been used as standard therapy in many patients with moderate or severe COVID-19 with oxygen demand because of its 28-day mortality reduction [4]. Tocilizumab is a monoclonal antibody against the Interleukin-6 receptor. Several RCTs have reported that suppression of inflammatory cytokines by IL-6 receptor antagonists may improve prognosis, based on a correlation between serum lL-6 levels and severity of COVID-19 [5]. The REMAP-CAP trial [6] showed that Tocilizumab reduced mortality in severe COVID-19 patients within 24 h of ICU admission who were on assisted ventilation (HFNC (FiO2 >0.4, Flow 30 L/min), NIV, intubation) or circulatory support (catecholamine administration). In the RECOVERY trial [7], tocilizumab was shown to reduce mortality in moderately ill COVID-19 patients with SpO2 < 92% or on oxygen and CRP ≥ 7.5 mg/dL.

Because both dexamethasone and tocilizumab decrease cellular immunity, their use can result in strong immunosuppression. A single-center, retrospective study [8] in Kenya reported that the secondary infection rate in COVID-19 patients (N = 913) was 17.2% in the tocilizumab-treated group and 4.8% in the non-treated group, with the rate being higher in the tocilizumab group. Although the underlying disease was different, a case report [9] of a patient with giant cell vasculitis who developed meningoencephalitis after treatment with tocilizumab, although the organism of origin was unknown. In another case report, an elderly male patient with COVID-19 who developed pneumococcal disease and died while receiving dexamethasone and tocilizumab was reported [10].

In a meta-analysis [2] of 24 studies, N = 3338, the frequency of bacterial co-infection at admission in COVID-19 was 3.5% and secondary infection 14.3%.

In a prospective multicenter cohort study [3] of COVID-19 hospitalized patients (N = 48 902 patients), the most common organisms as initiators of respiratory tract infection were Staphylococcus aureus and Haemophilus influenzae for co-infections at admission, and Staphylococcus aureus and Enterobacteriaceae for secondary respiratory tract infections. Bloodstream infections were most frequently caused by E. coli and Staphylococcus aureus.

In an observational study [11] in the United Kingdom, 32.7% of ICU patients developed concomitant bacterial infections, and 5.5% developed concomitant infections in the first 48 h after admission. Staphylococcus spp. and Streptococcus pneumoniae were more common, and Klebsiella pneumoniae and Escherichia coli contributed to the prolonged ICU stay. The authors reported that the rate of bacterial infections after 48 h of hospitalization was 27/1000 person-days, which was associated with an increased ICU mortality rate.

Catheter-related infection was initially the first differential in this case because Gram-positive cocci were isolated on blood culture. Subsequently, the organism of origin was found to be Streptococcus pneumoniae, so a spinal fluid puncture was performed, and a diagnosis of bacterial meningitis was made. The patient was in severe respiratory failure and continued to be managed under deep sedation, making it difficult to assess her level of consciousness and neurological evaluation. Meningitis was difficult to identify as the most likely diagnosis when the only symptom was fever. When a patient with severe COVID-19 fever develops, there is a wide range of differentials, including ventilator-associated pneumonia, catheter infection, and intra-abdominal infection, in addition to fever due to inflammation of COVID-19 itself. In addition, as a rule, the medical staffs must wear personal protective equipment (PPE), which raises the threshold for detailed physical examination, ultrasonography, and spinal fluid puncture to search for the source of fever in real practice.

In particular, when the patient is sedated, as in this case, neurological evaluation such as impaired consciousness is difficult, and the risk of delay in the diagnosis of bacterial meningitis is increased. Prompt diagnosis is necessary because delayed diagnosis and treatment of bacterial meningitis is associated with a high mortality rate and may worsen the neurological prognosis [12]. Bacterial meningitis should always be considered in COVID-19 patients who are immunosuppressed due to underlying disease or medications and who develop fever, especially if their level of consciousness is difficult to assess.

## Conclusions

Evidence for treatment of COVID-19 is accumulating. However, patients with COVID-19 can become immunocompromised by therapeutic agents. If an immunosuppressed COVID-19 patient is suspected of having a secondary infection, efforts should be made to identify the source of infection and bacterial meningitis should always be included in the differential.

## Ethical approval

The need for approval was waived by the Ethics Committee of Nakagami Hospital.

## Consent

Written informed consent was obtained from the patient for publication of this case report and any accompanying images.

## CRediT authorship contribution statement

**Yoshiaki Murayama:** Conceptualization, Writing – original draft. **Tomohiko Ishimine:** Writing – review & editing, Supervision. **Mikio Sasano:** Writing – review & editing. **Takafumi Todaka**: Data curation, Writing - review & editing. **Takashi Matsumoto:** Writing – review & editing. **Taiga Shimabukuro:** Writing – review & editing. **Risa Yonaha:** Writing – review & editing.

## Conflict of interest

The authors did not receive any specific grant from funding agencies in the public,commercial, or not-for-profit sectors.
